# Stability analysis for seed yield of chickpea (Cicer arietinum L.) genotypes by experimental and biological approaches

**DOI:** 10.18699/VJGB-23-19

**Published:** 2023-04

**Authors:** R. Karimizadeh, P. Pezeshkpour, A. Mirzaee, M. Barzali, P. Sharifi, M.R. Safari Motlagh

**Affiliations:** Kohgiloyeh and Boyerahmad Agricultural and Natural Resources Research and Education Center, Dryland Agricultural Research Institute, Agricultural Research, Education and Extension Organization (AREEO), Gachsaran, Iran; Lorestan Agricultural and Natural Resources Research and Education Center, Agricultural Research, Education and Extension Organization (AREEO), Khorramabad, Iran; Ilam Agricultural and Natural Resources Research and Education Center, Agricultural Research, Education and Extension Organization (AREEO), Ilam, Iran; Golestan Agricultural and Natural Resources Research and Education Center, Agricultural Research, Education and Extension Organization (AREEO), Gonbad, Iran; Department of Agronomy and Plant Breeding, Rasht Branch, Islamic Azad University, Rasht, Iran; Department of Plant Protection, Rasht Branch, Islamic Azad University, Rasht, Iran

**Keywords:** AMMI; HMRPGV, factorial regression (FR), mixed models, partial least squares regression (PLSR), simultaneous selection index (ssi), AMMI; HMRPGV, факторная регрессия (FR), смешанные модели, частичная регрессия наименьших квадратов (PLSR), индекс одновременной селекции (ssi)

## Abstract

A range of environmental factors restricts the production of chickpea; therefore, introducing compatible cultivars to a range of environments is an important goal in breeding programs. This research aims to find high-yielding and stable chickpea genotypes to rainfed condition. Fourteen advanced chickpea genotypes with two control cultivars were cultivated in a randomized complete block design in four regions of Iran during 2017–2020 growing seasons. The first two principal components of AMMI explained 84.6 and 10.0 % of genotype by environment interactions, respectively. Superior genotypes based on simultaneous selection index of ASV (ssiASV), ssiZA, ssiDi and ssiWAAS were G14, G5, G9 and G10; those based on ssiEV and ssiSIPC were G14, G5, G10 and G15 and those based on ssiMASD were G14, G5, G10 and G15. The AMMI1 biplot identified G5, G12, G10 and G9 as stable and high-yielding genotypes. Genotypes G6, G5, G10, G15, G14, G9 and G3 were the most stable genotypes in the AMMI2 biplot. Based on the harmonic mean and relative performance of genotypic values, G11, G14, G9 and G13 were the top four superior genotypes. Factorial regression indicated that rainfall is very important at the beginning and end of the growing seasons. Genotype G14, in many environments and all analytical and experimental approaches, has good performance and stability. Partial least squares regression identified genotype G5 as a suitable genotype for moisture and temperature stresses conditions. Therefore, G14 and G5 could be candidates for introduction of new cultivars.

## Abstract (ru)

Ряд факторов окружающей среды ограничивают производство нута, поэтому исследование сортов в различных средах является важной составляющей селекционных программ. Цель представленного исследования заключалась в поиске высокоурожайных и устойчивых к условиям богарного земледелия генотипов нута. Четырнадцать перспективных генотипов нута с двумя контрольными сортами выращены в рандомизированном полном факторном эксперименте в четырех регионах Ирана в вегетационные периоды 2017–2020 гг. Первые два главных компонента объясняют 84.6 и 10.0 % генотипа взаимодействиями с окружающей средой (GEI) соответственно. Лучшими генотипами на основе индексов одновременной селекции ASV (ssiASV), ssiZA, ssiDi и ssiWAAS стали G14, G5, G9 и G10, на основе ssiEV и ssiSIPC – G14, G5, G10 и G15, на основе ssiMASV – G14, G5, G10 и G15. Результаты AMMI1-биплот-анализа позволили идентифицировать G5, G12, G10 и G9 как стабильные и высокоурожайные генотипы. По данным модели AMMI2-биплот наиболее стабильными определены генотипы G6, G5, G10, G15, G14, G9 и G3. На основе гармонического среднего и относительной эффективности генотипи- ческих значений (HMRPGV) G11, G14, G9 и G13 отмечены как четыре лучших генотипа. Факторная регрессия показала, что количество осадков крайне важно в начале и конце вегетационного периода. Генотип G14 проде- монстрировал хорошую урожайность и стабильность во многих различных условиях среды и при использова- нии всех аналитических и экспериментальных подходов. Методом частичной регрессии наименьших квадратов генотип G5 был идентифицирован как наиболее устойчивый к неблагоприятным условиям – как по влажности, так и по температуре. Следовательно, G14 и G5 могут быть кандидатами для интродукции новых сортов.

## Introduction

Chickpea (Cicer arietinum L.) is one of the most important pulse crops that are well adapted to arid and semiarid conditions. Pulse crops are important sources of protein in human food and are suitable for animal feeds (Gaur et al., 2010). Chickpea is the fifth most rainfed crop in Iran and its harvested area is about 561,000 ha in Iran, which is mostly (98.59 %) cultivated in dryland areas (FAO, 2020) FAO. Statistics of Food and Agriculture Organization. 2020. https://www.fao. org/statistics/en/. Chickpea is a cool season legume and sensitive to heat stress (Devasirvatham et al., 2012) grown mainly in semi-arid and arid regions, where its production is restricted by a range of environmental factors such as high (or very low) temperature, lack (or excess) of soil moisture availability and day length (Richards M.F. et al., 2020).

Introducing compatible cultivars to a range of environments is the important goal in breeding programs (Karimizadeh, Mohammadi, 2010). Awareness of genotype by environment interaction (GEI) helps breeders to check genotypes more accurately and select the best genotypes. Because of exhibition of various phenotypic expressions of a specified genotype to different environments and unknown responses of some of the genotypes to a specified environment, investigation of GEI depends on the phenotypic stability and adaptation of genotypes (Yan et al., 2000). In other words, GEI created a hard situation for breeders and growers to choose highyielding and stable varieties to different environments and decreased the efficiency in selection of superior genotypes and cultivar introduction (Yan, Kang, 2003). Because a stable variety is adapted to environmental variation, plant breeders are interested in the analysis of yield stability as a worthwhile characteristic of a genotype (Annicchiarico, 2002). Therefore, for evaluation of yield stability and performance, it is very important to use a variety to wide range of environments (Yan et al., 2011). Developing broadly adapted genotypes with a high level of phenotypic stability and yield potential is a tool to overcome the genotype by environment interaction (Kanouni et al., 2015). However, since it is difficult or impossible to find such a variety, specific adaptation of varieties permits plant breeders to manage GEI and develop suitable genotypes for different environments (Gauch, Zobel, 1997).

Statistical models, which incorporate environmental and genotypic variables into the multi-environmental trial (MET) analysis, have been used to study and explain GEI. Two main statistical methods for analyzing GEI are experimental (or empirical) and analytical (or biological) approaches (van Eeuwijk et al., 1996). The empirical approaches focus on performance-based selection, whereas the analytical approaches refer to the integration of some agronomic/climatic variables that determine the response variable (such as grain yield) (Richards R.A., 1982). Factorial regression (FR) (van Eeuwijk et al., 1996) and partial least squares regression (PLSR) (Vargas et al., 1998), which directly incorporate environmental variables and/or external varieties, can be considered as a predictive strategy for recommendation purposes (Basford, Cooper, 1998).

There are several methods for stability analysis in experimental approaches, including multivariate and univariate models. Additive main effect and multiplicative interaction (AMMI) (Gauch, Zobel, 1988), as a multivariate model in experimental approach, is postdictive, because it has to handle the problem of repeatability of GEI (Basford, Cooper, 1998). All models attempt to provide a biological interpretation of GEI using information on external environmental and/or external genotypic variables. An alternative method of experimental approach for stability analysis is the harmonic mean, and the relative performance of genotypic values (HMRPGV) based on mixed models (Resende, 2007). This method provides information on stability, adaptability and yield performance of genotypes in the same unit and scale. In this method, selection of the genotypes with the highest values of harmonic mean of genotypic values (HMGV), relative performance of genotypic values (RPGV) and HMRPGV allows a simultaneous selection for yield performance and stability. This methodology is used in evaluation of stability of yield performance in rice (Colombari-Filho et al., 2013), wheat (Coan et al., 2018; Verma, Singh, 2020) and corn (Rodovalho et al., 2015). M.A. Rodovalho et al. (2015) compared HMRPGV, Lin and Binns’s and Annichiarico’s methods for stability of maize hybrids and indicates high agreement between these methodologies, however, the HMRPGV method enables breeders to directly assess the breeding values for the yield, genotypic stability and adaptability simultaneously. The FR has been used successfully to interpret GEI in maize (Romay et al., 2010), wheat (Campbell et al., 2004; Voltas et al., 2005; Joshi et al., 2010), durum wheat (Mohammadi et al., 2020a, b) and barley (Ahakpaz et al., 2021). PLS regression to interpret the GE interaction has also been applied in wheat (Vargas et al., 1999; Kondić-Špika et al., 2019), maize (Stojaković et al., 2015), sorghum (Das et al., 2012) and barley (Hilmarsson et al., 2021). Although many researchers have evaluated stability of chickpea genotypes by stability methods such as AMMI (Farshadfar et al., 2011, 2013; Zali et al., 2012; Funga et al., 2017; Pouresmael et al., 2018; Azam et al., 2020), there have been no reports of analytical approaches in the case of this crop.

This study was carried out to get high-yielding and adaptable genotypes to rainfed condition of Iran, to compare empirical methods and to assess the role of climatic factors in GEI.

## Materials and methods

Experimental conditions and plant material

Fourteen advanced chickpea genotypes with two control varieties (Adel and Azad) (Table 1) were cultivated in randomized complete block design in four regions of Iran, including Gachsaran, Gonbad, Khorramabad and Ilam (Table 2), during 2017–2020 growing years. The experiment was performed in Gonbad in every three cropping years, in Gachsaran and Ilam in the first two cropping years and in Khorramabad only in the second cropping year. Fifty seeds per m2 were grown in plots with six m length and one m wide. Chemical fertilizer at the rate of 100 kg ha–1 of ammonium phosphate and 35 kg ha–1 of urea was evenly mixed with the soil. After harvest, seed yield was weighed and statistical analyzes were performed on the data.

**Table 1.**
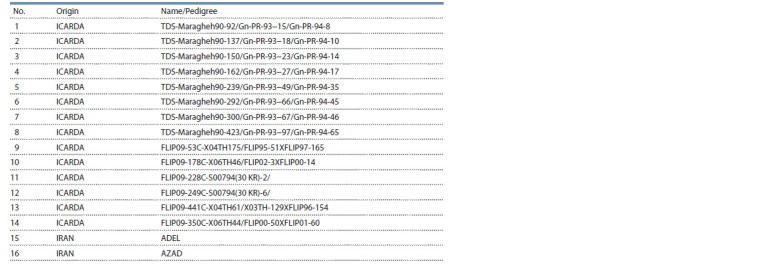
Code and name of studied chickpea genotypes

**Table 2.**
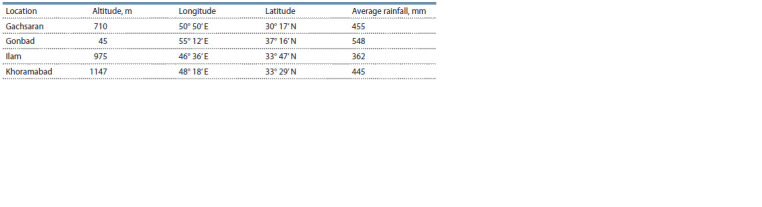
Geographic characteristics of trials area

Statistical analysis

Experimental approaches. The AMMI model was used to analyze the genotype (G) × environment (E) interactions. AMMI constitutes a model family, with AMMI0 having no interaction principal component (IPC), AMMI1 having 1 IPC, AMMI2 having 2 IPC, and so on up to AMMIF (residual discarded). The AMMI model equation is:

**Formula:**
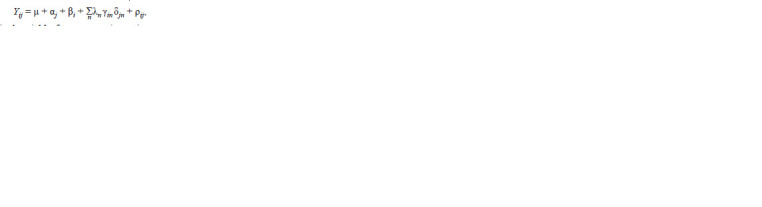
Formula

where Yij is the yield of genotype i in environment j; μ is the grand mean; αi is the genotype deviation from the grand mean; βj is the environment deviation; λn is the singular value for IPC n and correspondingly λ2 n is its eigenvalue; γin is the eigenvector value for genotype i and component n; δjn is the eigenvector value for environment j and component n, with both eigenvectors scaled as unit vectors; and ρij is the residual.

Simple and combined analysis of variance and stability analysis performed by METAN R packages (Olivoto, DalCol Lucio, 2020). The agricolae R package (Mendiburu, 2019) was also used for calculation of some of AMMI indices. Stability indices were calculated using the equations in Table 3.

**Table 3.**
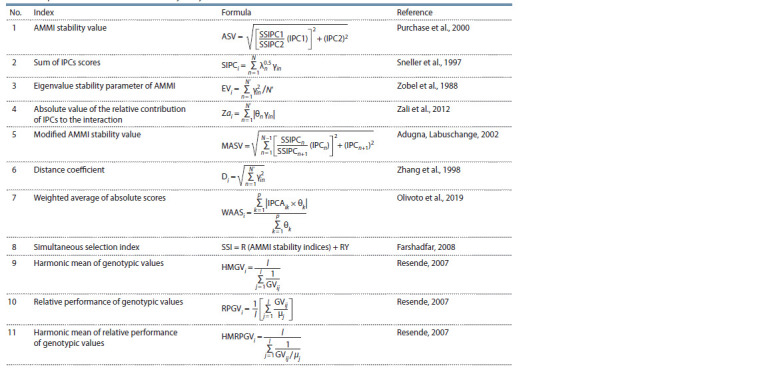
Equations for calculation the stability analysis indices

The SSIPC1/SSIPC2 ratio in equation 1 is the weight assigned to the first interaction principal component (IPC1), which is the product of dividing the sum of squares of first IPC by the sum of squares of the second IPC. In equation 2, λn is the root of the nth IPC, which for SIPC1 and SIPCF is one and the number of principal components remaining in the model, respectively. In equations 3 and 4, γin is the root of the nth axis and N ′ is the number of significant principal components in the analysis of variance of AMMI by F-test. In equation 4, the percentage of the sum of squares explained by the nth axis of IPC denotes by θn. In equation 5, SSIPC1, SSIPC2, …, SSIPCn are the sum of squares of the 1st, 2nd, ..., and nth IPC; and PC1, PC2, …, PCn are the scores of 1st, 2nd, ..., and nth IPC. In equation 6, the AMMI distance (D) calculated as distance of the interaction principal component from the origin. In equation 7, IPCik is the score of i th genotype on k th IPC axis. θk is the explained variance of the k th IPCA for k = 1, 2, …, p, considering p the number of significant PCAs. In these equations, the most stable genotypes have the lowest values of stability indices.

In equation 8, the simultaneous selection index (ssi) is the sum of the rankings of genotypes based on the AMMI [R (AMMI stability indices)] and the average rank of seed yield of genotypes in all environments (RY). AMMI1 (IPC1 vs. seed yield) and AMMI2 (IPC1 vs. IPC2) biplots were drawn using the standard method described by R.W. Zobel et al. (1988).

The BLUP model for MET trials, unlike the classical additive model, assumes the genotypic effects as random and uses a different computational procedure (Olivoto et al., 2019). In BLUP, μj is the general mean for j th environment; l is the number of environments; GVij: uj + gi + geij is the genotypic value of i th genotype in j th environment. uj is the mean of the j th environment, and gi and geij are the BLUP values of i th genotype and the interaction between i th genotype and j th environment, respectively. Stability indices based on this mixed model are: HMGV, RPGV and HMRPGV were calculated by Equations 9–11, respectively (Table 3).

Analytical approaches. Seasonal rainfall and average temperature of autumn, winter and spring were used as environmental co-variables. Integration of external data into GEI analysis by PLSR and FR methods was carried out by GEA-R software (Pacheco et al., 2015). Partial least squares regression The PLSR model includes independent matrices X (rainfall and average temperature data) and a dependent matrix Y (seed yield) and the latent variables t as follows (Vargas et al., 1998):

**Formula 1.**
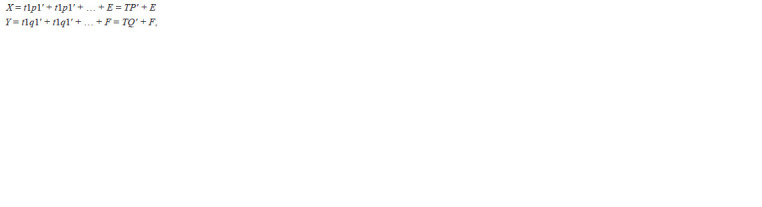
Formula1

where, matrices T, P and Q contain X-scores, X-loadings and Y-loadings, respectively. F and E are the residuals of the unexplained variation. A biplot was built based on the first two PLSR factors to investigate the relationships among covariables, genotypes and environments

Factorial regression The FR model is also as follows (van Eeuwijk et al., 1996):

**Formula 2.**
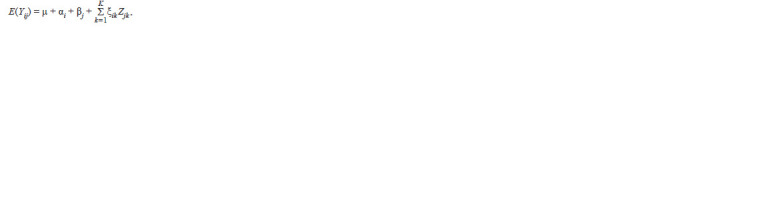
Formula2

the value of any environmental variable k for environment j; and ξik represents the sensitivity of genotype i to the explicit environmental variable k. The heterogeneity in the ξi’s for successive z1... zK variables accounts for the interaction, while the sum of multiplicative terms KΣ k = 1 ξik Zjk approximates GE. To facilitate the interpretation of genotype by environment, the external variables can be centered to mean zero. The parameter ξik can be easily estimated by standard least squares techniques

The Akaike’s information criterion (AIC) (Akaike, 1974) was used to determine the number of covariates that are included in the model.

## Results

Analysis of variance

Analysis of variance showed that the effects of environment, genotype and genotype by environment interaction were significant on seed yield at 1 % probability level and these three components explained 37.13, 16.90 and 31.30 % of phenotypic variation, respectively (Table 4).

**Table 4.**
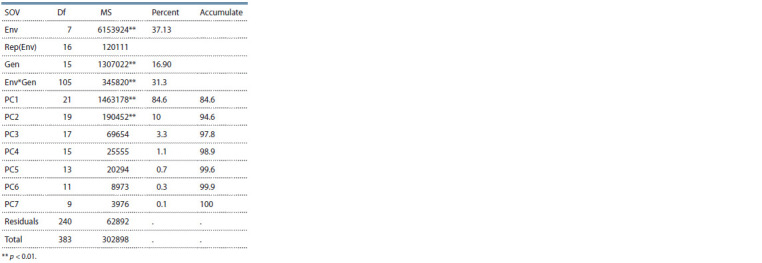
AMMI analysis of variance for seed yield of chickpea genotypes

Experimental stability approaches

AMMI stability indices and ssi. The highest seed yield was observed in G11, followed by G14, G9, G16 and G13, which were higher than average yield of genotypes in all environments (1069.25 kg ha–1). Stability of genotypes was evaluated across different environments by AMMI indices. The ASV, WAAS, Za and MASV stability indices identified genotypes G5, G14, G12, G1 and G10 as the most stable genotypes. The SIPC and EV indices indicated genotypes G14, G5, G6, G10 and G15 were the most stable genotypes. According to D index, genotypes G14, G5, G10, G6 and G1 were more stable than other genotypes (Table 5).

**Table 5.**
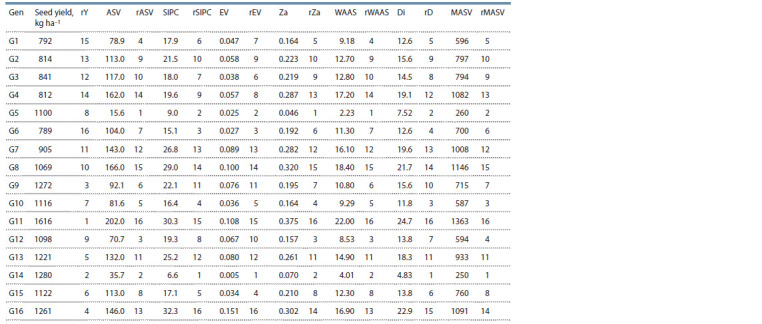
AMMI-based stability indices and ranks for stability indices Note. ASV, AMMI stability value; SIPC, sum of IPCs scores; EV, eigenvalue stability parameter of AMMI; Za, absolute value of the relative contribution of IPCs to the interaction; WASS, weighted average of absolute scores.

Based on ssi of AMMI stability value (ssiASV), ssiZA, ssiDi and ssiWAAS, genotypes G14, G5, G9, G10 and G12 were identified as superior genotypes; while based on ssiEV and ssiSIPC, genotypes G14, G5, G10, G15 and G9 were the superior genotypes. The ssiMASV index identified genotypes G14, G5, G10, G15 and G12 as superior genotypes (Table 6).

**Table 6.**
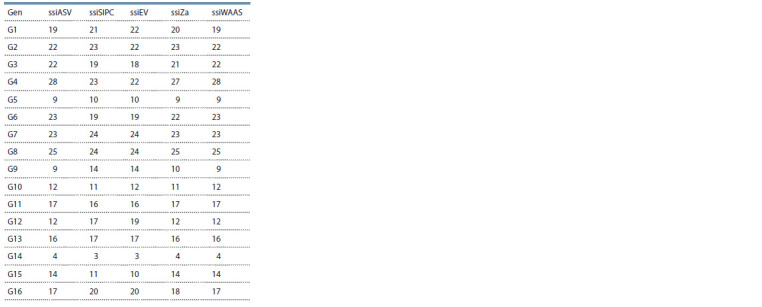
Simultaneous selection indices (ssi) for each of genotypes

**Fig. 1.**
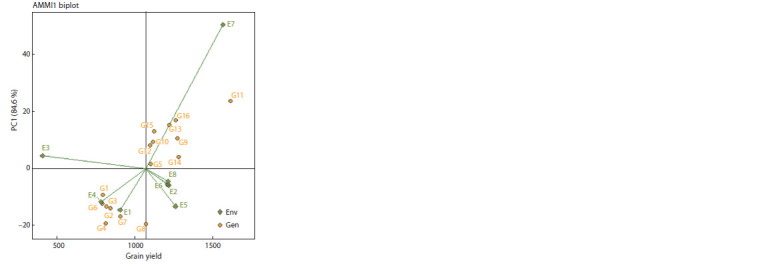
AMMI1 biplot for identity of the superior lentil genotypes based on seed yield mean and PC1. The naming of genotypes is similar to Table 9. E1, Gachsaran 2017-18; E2, Gonbad 2017-18; E3, Ilam 2017-18; E4, Gachsaran 2018-19; E5, Khorramabad 2018- 19; E6, Gonbad 2018-19; E7, Ilam 2018-19; E8, Gonbad 2019-20.

Biplot interpretation. The AMMI1 biplot indicated the score of the first principal component in genotypes G5, G12, G10, G9, and G14 was near zero and so these genotypes had low interaction with environment and were identified as stable genotypes. The yield of these genotypes was also higher than the average seed yield of all genotypes in all environments (1069.25 kg ha–1). Genotypes G11, G8, G16, and G4 at the farthest point from the biplot origin were unstable genotypes (Fig. 1).

**Fig. 2.**
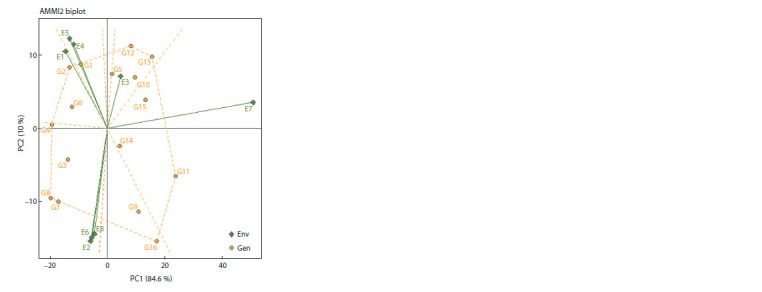
The AMMI2 biplot for identity of the superior chickpea genotypes based on first PCs. The naming of genotypes and environments is similar to Table 9 and Fig. 1, respectively

The AMMI2 biplot showed that genotypes G4, G2, G1, G12, G13, G11, G16, G7 and G8 with the longest distance from the biplot origin had a high contribution in genotype by environment interaction and were unstable genotypes, but these genotypes adapted to their close environments (Fig. 2). Therefore, genotype G2 was the best genotype for E1; genotype G1 for E4 and E5; genotypes G12 and G13 for E3; genotype G11 for E7 and genotypes G7 and G8 for E2, E6 and E8. The other genotypes within polygon, including G6, G5, G10, G15, G14, G9 and G3, were the most stable genotypes. Other usefulness of this biplot, in addition to identifying adaptable genotypes with any environment and introducing genotypes with general stability, are identification of environments with the long vector that could be more effective in finding stable genotypes (Yan, Kang, 2003). Accordingly, all environments except for E3 could be used as discriminative and representative environments

Determination of genotypic stability and adaptability using HMRPGV. The top four superior genotypes compared to control varieties (ADEL and AZAD), based on the measure of stability and adaptability (HMRPGV), were genotypes G11, G14, G9 and G13. The products of this index and the general mean (HMRPGV*μ ) of these genotypes were 1570, 1287, 1231 and 1200 kg ha–1, respectively (Table 7). The selection of these genotypes for seed yield increased to 20.63 % over the general mean (1069.25 kg ha–1).

**Table 7.**
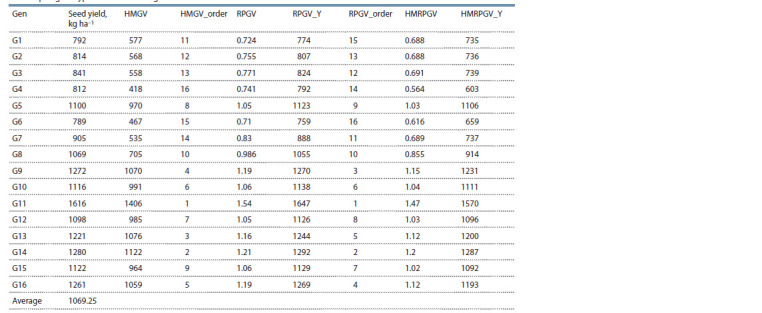
Ranking of the genotypes in all environments evaluated for adaptability parameters of genotypic values for the grain yield of chickpea genotypes evaluated in eight environments Note. RPGV, performance genetic value; HMGV, harmonic mean of genotypic values; HMRPGV, harmonic mean and relative performance of genotypic values.

Analytical stability approaches

FR analysis. Since the climatic information of the third year in Gonbad was not available, analytical approaches (factorial and partial least squares regression) with environmental covariables were performed with seven environments. The steps for climatic variables in the FR model based on AIC indicated average temperature in autumn (FallT), average rainfall in spring (SpringR), average rainfall in autumn (FallR) and average temperature in spring (SpringT) were detected to be important contributors to GE interaction (Table 8).

**Table 8.**
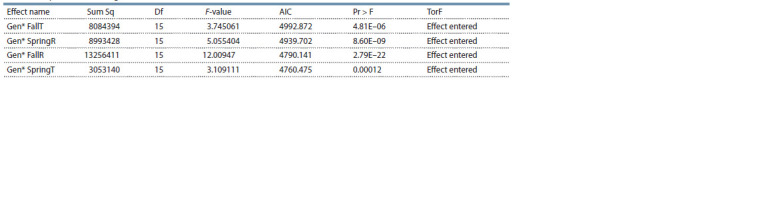
Stepwise factorial regression model for climatic variables based on Akaike’s information criterion (AIC) Note. FallT, average temperature in autumn; SpringT, average temperature in spring; SpringR, average rainfall in spring; FallR, average rainfall in autumn

**Fig. 3.**
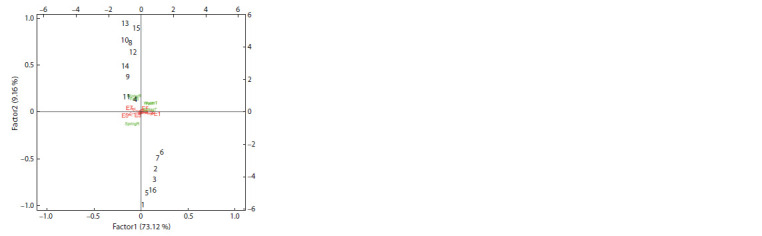
The biplot based on PLSR method with rainfall seasons’ covariates for seed yield of 16 chickpea genotypes in seven environments. FallT, average temperature in autumn; winterT, average temperature in winter; SpringT, average temperature in spring; SpringR, average rainfall in spring; winterR, average rainfall in winter; FallR, average rainfall in autumn. The naming of genotypes and environments is similar to Table 9 and Fig. 1, respectively

Partial least squares regression analysis. The first and second PLSR factors based on environmental co-variables accounted for 73.12 and 9.16 % of the GE interaction sum of squares, respectively (Fig. 3). Environments located on the right-hand side of the biplot (E1, E2, E4 and E6) had high values for temperature co-variables (i. e., FallT, SpringT and WinterT), whereas the other environments (E3, E5 and E7) on the opposite side tended to have high rainfall (Table 9). These results indicate that some genotypes (G4, G11, G9, G14, G12, G8, G10, G15 and G13) performed better in high rainfall in winter and autumn seasons (see Fig. 3). The PLSR biplot displayed that high rainfall in the environments in the west of Iran (E3, Ilam 2017-18; E5, Khorramabad 2018-19 and E7, Ilam 2018-19) led to high performance in genotypes

**Table 9.**
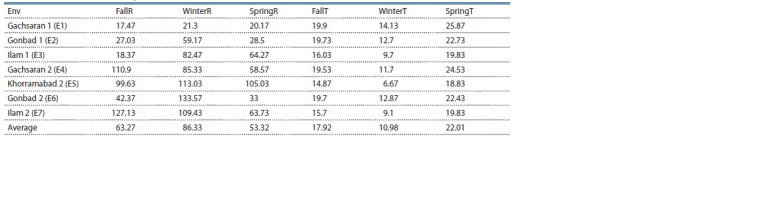
Seasonal rainfall and temperature in seven environments Note. The naming of environments is similar to Fig. 1.

## Discussion

The significant effect of genotype (16.90 %) and environment (37.13 %) is a sign of the comprehensive genetic background of experimental materials and diversity of experimental locations and cropping seasons. The significant effect of GEI shows different performance of genotypes in different environments. Other researchers also reported a greater contribution of environmental effect on total variation of chickpea seed yield (Farshadfar et al., 2011; Sayar, 2017; Pouresmael et al., 2018; Azam et al., 2020). Therefore, due to dependence of diversity of seed yield of chickpea genotypes on environment and genotype by environment interaction, further analysis needs to increase the selection efficiency of genotypes. In other words, the presence of significant GEI indicates the necessity to find the yield potential and adaptability of genotypes based on evaluations at several locations and in cropping seasons (Annicchiarico, 2002). Since the genotype by environment interaction can reduce any improvement due to selection, therefore, in selection of cultivars, combination of stability with seed yield can lead to good results (Yan et al., 2001).

AMMI analysis of variance showed a high contribution of the first two principal components, especially PC1 (84.6 %) in GEI. Other researchers also indicated contribution of 52.5 and 21.95 % (Tilahun et al., 2015), 40.5 and 27.5 % (Farshadfar et al., 2013), 56.0 and 24.0 % (Farshadfar et al., 2011), 53.34 and 33.25 % (Azam et al., 2020) and 32.7 and 20.4 % (Funga et al., 2017) of the first two principal components in GEI of chickpea seed yield. In accordance with the results of present study, the other researchers were also identified stable chickpea genotypes by AMMI stability indices (Farshadfar et al., 2011, 2013; Zali et al., 2012; Funga et al., 2017; Pouresmael et al., 2018). Since the first two principal components had a high contribution on genotype by environment interaction, the stability indices including ASV, WAAS, Za and MASV had similar results in identifying the stable genotypes

Identification of superior genotypes with AMMI indices was only based on genotype stability; so, genotypes G1 and G6 with a lower yield than average seed yield were identified as stable genotypes. Hence, ssi (Farshadfar, 2008) based on AMMI indices was used to find the superior genotypes. Since both aspects of stability and yield of a genotype were used in simultaneous selection index, the use of these indices prevents selection of stable genotypes with a low yield (Farshadfar, 2008). In accordance with these findings, A. Funga et al. (2017) also used ssi for yield performance and stability in chickpea to find stable and high-yielding genotypes. Use of simultaneous selection index for yield performance and stability can perform selection process with more confidence (Moghadam, 2003).

Based on the AMMI1 biplot, G5, G12, G10, G9, and G14 were the stable genotypes. Because the AMMI1 biplot uses both aspects of stability based on the first principal component and seed yield to identify stable genotypes, when the contribution of the first principal component in GEI is high (84.6 %), the results of the AMMI 1 biplot are very similar to the results of ssi based on AMMI indices. H.G. Gauch and R.W. Zobel (1988) stated that despite the high value of environment main effect, for evaluation of genotypes, only the effects of genotype (G) and GEI are appropriate and so it is necessary to remove the environment mean effect (E) and concentrate on G and GE.

The AMMI2 biplot identified G6, G5, G10, G15, G14, G9 and G3 as the most stable genotypes. This view of the biplot was also used for identifying the adapted genotypes to any of environments, so that the genotype placed at the top of each section is the best genotype for the environments in that section (Yan et al., 2000). Genotypes G7 and G8 ware compatible with three environments E2, E6 and E8; they can be considered as genotypes with specific adaptability to these environments. Identification of environments with the long vector could be more effective in finding stable genotypes (Yan, Kang, 2003). The discrimination and representatives of all of the environments except E3 must be ascribed to the amount of rainfall and its proper distribution in different seasons. In agreement with the present finding, other researchers have identified stable chickpea genotypes using the AMMI2 biplot (Pouresmael et al., 2018; Funga et al., 2017; Farshadfar et al., 2013). Another remarkable point is that when the contribution of the first principal component is very high, identification of stable and high-yielding genotypes based on the AMMI1 biplot is better than on the AMMI2 biplot, so that G12, which was unstable in the AMMI2 biplot, in terms of ssiASV, ssiZA, ssiDi and ssiWAAS and AMMI1 was found to be the superior genotype

The top four superior genotypes compared to control varieties based on HMRPGV were G11, G14, G9 and G13. In HMRPGV, the predicted genotypic values are declared as a proportion of the overall mean for each environment and then the mean value of this ratio is obtained across the environments (Rodovalho et al., 2015). The selection of genotypes in this method is based on stability, adaptability and yield performance; therefore, this method indicated a positive response of genotypes to environmental improvements and the stability of genotypes over the environments. M.D.V. Resende (2007) declared the HMRPGV method evaluated simultaneously seed yield, adaptability and stability, in a genotypic context. In this stability index, the genotypes can be simultaneously sorted by genotypic values and stability using the harmonic means of the BLUP (Rodovalho et al., 2015).

The analytical approach to analyzing GE interaction is important to enhance the value of MET and gain an understanding of the causes of GE interaction. These approaches have been demonstrated successfully in a range of crop species (van Eeuwijk et al., 1996; Mohammadi et al., 2020a, b). Factorial regression indicated rainfall to be very important at the beginning of the season to germination and establishing of seedlings and at the end of the season for its proper developmental and reproductive growth stages. In confirmation of this result, S.H. Sabaghpour et al. (2006) stated that chickpea needs the most water during flowering, podding and seed filling and so, due to the lack of rainfall during these stages, terminal drought stress is a major abiotic stress for reducing chickpea productivity.

The rainfall was relatively high in environments E3, E5 and E7 that favored the positive GE interaction with G13, G15, G10, G8, G12, G14, G9 and G4. The best genotypes based on experimental methods (G11, G14, G9, G13, G5, G10, G15 and G12) were in the upper left quarter of the PLSR biplot (see Fig. 3). The seasonal rainfall of autumn and winter in environments E3, E5 and E7 in this quarter of the biplot, especially the last two environments, were higher than the average seasonal rainfall of all environments. The average seasonal temperature was also lower than the average temperature of all environments in these three environments. Thus, these environments can be considered as favorable environments in terms of these two climatic co-variables and the mentioned genotypes (G11, G14, G9, G13, G5, G10, G15 and G12) can be identified as superior genotypes in favorable conditions. The AMMI2 biplot also identified genotype G11 as a desirable genotype for environment E7.

On the other hand, the seasonal rainfall in environment E1 was much lower than the average seasonal rainfall in all environments, and its temperature was higher than the seasonal temperature in all environments. Therefore, this trial environment can be considered as an environment with drought and heat stresses for chickpea, which is a cold-loving crop. The PLSR biplot also demonstrates this hypothesis well, because the seasonal rainfall was on the opposite side of this environment and the average seasonal temperature was on its same side. Hence, genotypes located in the quadrant of this environment (right and bottom) can be considered genotypes tolerant to drought and heat stresses. The AMMI2 biplot also identified genotypes G2 and G6 as suitable for this environment. This environment had a high discriminating power due to the vector length in the AMMI2 biplot, so its results can be trusted and these results can be used properly. In the PLSR biplot, genotypes G6, G7, G2, G3, G16, G5 and G1 were in the same quarter along with environment E1. From these genotypes, G5 and G16 had a higher performance than the average yield of all genotypes and can be considered as tolerant genotypes to heat and drought stresses. Since genotype G16 was previously introduced as a cultivar, genotype G5 can be recommended as a suitable genotype for dryland and hot conditions. Such a conclusion is possible only from a combination of analytical and experimental approaches. If such analyzes were not performed here, we would not be able to achieve such results. It is happening on moisture stress towards the end of the cropping season with frequent events of heat stress in chickpea. Thus, the crop is exposed to stress conditions during the reproductive stage causing yield losses (Devasirvatham, Tan, 2018). A decrease in chickpea yields was observed with a 1 °C increase in seasonal temperature (Kalra et al., 2008; Upadhaya et al., 2011). Similarly, with every 0.1 °C temperature rise combined with 31 % reduction in seasonal rainfall, the yield of chickpea decreased (Dubey et al., 2011). This shows that high temperature and drought are the major factors that affect chickpea production. M.D. Kadiyala et al. (2016) have stated that unpredictable climate change is the main restriction for chickpea production as it increases the frequency of drought and temperature extremes, i. e. high (> 30 °C) and low (< 15 °C) temperatures, which reduces seed yield considerably. Thus, high and stable yielding cultivars of chickpea during such stress conditions need to be developed (Chaturvedi, Nadarajan, 2010; Krishnamurthy et al., 2010; Devasirvatham et al., 2015; Devasirvatham, Tan, 2018).

## Conclusion

Stability analysis was performed by analytical (FR and PLSR) and experimental (AMMI analysis and HMRPGV based on mixed models) approaches. Simultaneous selection index was superior to AMMI indices for identifying stable and high yielding genotypes. Comparison between HMRPGV method and AMMI indices shows that HMRPGV index relies more on seed yield performance than stability of the genotype, so that genotypes G11 and G13, which were not stable in any of the AMMI indices and had specific adaptation to environments E7 and E3, respectively, with HMRPGV stability index, have been identified as superior genotypes. Factorial regression indicated that rainfall is very important at the beginning and end of the growing seasons. The PLSR biplot indicated that E3, E5 and E7 can be considered as favorable environments in terms of seasonal rainfall and temperature and G11, G14, G9, G13, G5, G10, G15 and G12 can be identified as superior genotypes in favorable conditions. In general, based on different methods, genotype G14 had good performance and stability of seed yield in many environments and in all of the methods and could be a candidate for introduction of new cultivars. The PLSR biplot also identified genotype G5 as a suitable genotype for moisture and temperature stresses conditions.

## Conflict of interest

The authors declare no conflict of interest.
